# Structural and adsorption behaviour of ZnO/aminated SWCNT-COOH for malachite green removal: face-centred central composite design

**DOI:** 10.3906/kim-2011-38

**Published:** 2021-08-27

**Authors:** Zeynep CİĞEROĞLU

**Affiliations:** 1 Department of Chemical Engineering, Faculty of Engineering, Uşak University, 64300, Uşak Turkey

**Keywords:** Malachite green adsorption, optimization, ZnO/aminated SWCNT-COOH nanocomposite, response surface methodology

## Abstract

In this study, an effective adsorbent was synthesized to remove malachite green (MG), which is one of the toxic dyes. Firstly, single walled carbon nanotube with carboxylated acid (SWCNT-COOH) was functionalized with diethylenetriamine and a new nanocomposite was obtained using nano zinc oxide (ZnO) powder. The effects of pH (3–7), the amount of adsorbent (5–15 mg) and the initial concentration (10–50 mg L^–1^) of the solution on the adsorption uptake were investigated. The optimal parameters that maximize the adsorption uptake according to the specified working range are found to be 4.63 for pH, 49.94 mg L^–1^ for initial concentration, 5.25 mg for the adsorbent dose, and the maximum adsorption capacity has been found as 52.26 mg g^–1^. The excellent fitting of the pseudo-second kinetic model with (R^2 ^= 0.9912) was fitted the experimental data. The Freundlich isotherm model gave a clue about the type of adsorption. Furthermore, thermodynamic results showed that adsorption process was endothermic.

## 1. Introduction

There are more than 100,000 commercial dyes registered in the world every year, and over 7 x 10^5^ tons of synthetic dyes are produced [1,2]. Some paints that cannot be used and are discharged as industrial wastewater. These wastewater effluents into the lakes, rivers and soil and harms the entire ecosystem. One of these synthetic dyes is malachite green (MG), known to be classified among the triphenylmethane dyes. MG is also used in jute, silk, cotton, ceramic, wool, and leather dyeing [3]. In the literature, MG with a concentration of less than 0.1 µg mL **^–^** ^1^ has been reported to be highly toxic to mammals [4]. In some mammals, MG has been observed to cause mutagenic, carcinogenic, lung tumor formation and reproductive abnormalities [5]. Photocatalytic [6], the Fenton process [7], and adsorption processes [8] are available in the literature for the elimination of malachite green, which harm to the living health and ecosystem, from wastewater. Among these processes, adsorption is more preferred for its economic and easy processing.

In a literature survey for MG adsorption from aqueous solutions, Hameed and El-Khaiary synthesized rattan shavings [9]. Besides, Hameed and Khaiary prepared the adsorbent obtained by derivatizing the rice straw [10]. Hameed and Khaiary also synthesized activated carbon obtained from the bamboo activated with potassium carbonate was used for treatment MG [11]. Apart from that, Yildirim and Bulut synthesized composites using chitosan/polyacrylic acid and bentonite [12] and observed the adsorption behaviour of malachite green onto their composites.

Response surface methodology (RSM) is a combination of algebraic functions and statistical analysis approach, which provides the determination of optimal variables as well as maximal/minimal response. While presenting the quadratic (well-fitted) equation, it serves as an optimization technique [13].

Recently, notably nanomaterials are used as adsorbents to remove dye pollutants from wastewater [14]. Since nano zinc oxide and carbon nanotubes have a high surface area, they are recently preferred in adsorption studies [15]. Moreover, nano zinc oxide can be used as an adsorbent because of nontoxic, cheap, and stable.

According to the literature research, no study has been found on the elimination of the MG from the aqueous media of the adsorbent synthesized using zinc oxide with functionalized SWCNT-COOH. This study investigated the adsorption uptake of the synthesized adsorbent on the MG from aqueous media. In addition, pH, initial concentration of MG and the amount of adsorbent were considered as variables affecting the adsorption process. Generating 3-D graphics (according to the maximum response) show the synergistic relationships between the factors. In addition, kinetic studies were conducted to discover the adsorption mechanism. Isotherm studies were carried out to determine the relationship between the adsorbent and adsorbate. 

## 2. Materials and methods

SWCNT-COOH (65% C basis) is supplied from Nanografi, ODTU Teknopark, Ankara. Malachite green, dietyhlenetriamine, and NaNO_2 _are supplied from Sigma-Aldrich.

### 2.1. Preparation of nanocomposite

To functionalize SWCNT-COOH with diethylenetriamine, a slightly modified of Chidawanyika and Nyokong’s method was applied [16]. Briefly, 140 mg of SWCNT-COOH was transferred to the Erlenmeyer flask and 2 mL of diethylenetriamine in 25 mL of distillated water was added. Then, 0.194 g of NaNO_2_ was added slowly to the flask. For almost 150 min, the mixture was placed on a magnetic stirrer at the speed of 400 rpm and 60 ºC. The mixture was removed from a magnetic stirrer and the material was separated by nuche filtration. Then, distilled water was used to wash the material until the neutral pH (~7).

### 2.2. Characterization analysis of nanocomposite

To determine the functionality of the nanocomposite, FTIR spectrometer (Bruker Vertex 70) was used by using the KBr pellet technique. The crystalline structure of the nanocomposite was evaluated via X-ray diffraction. Scanning electron microscopy (SEM) was applied to the surface structure of nanocomposite. 

### 2.3. Batch adsorption experiments via statistical design

Adsorption experiments were applied according to face-centred central composite design (FCCCD) using Design Expert Trial Version 12. FCCCD has star points that are within central levels [17,18]. In this study, three levels of parameters such as pH (3–7), adsorbent dose (5–15 mg), and initial concentration of MG (10–50 mg L^–1^) were selected. The alkaline medium was not chosen because the MG is not stable [19]. MG concentration was determined by ultraviolet-visible (UV-vis) spectrophotometer (Perkin Elmer, Lambda 365) at 618 nm before and after adsorption tests. Eq. (1) shows how to calculate the adsorption of nanomaterial.

(1)qe=Co-CemxV

In Eq. (1), V is represented volume of solution (L), C_o_ is the initial concentration of MG (mg L^–1^), Ce is the equilibrium concentration of solute after the adsorption process (mg L^–1^), m is mass of adsorbent (g).

### 2.4. Kinetic models

In order to decide the adsorption equilibrium, adsorption kinetics should be applied. Adsorption rates and adsorption mechanism can be evaluated in terms of appropriate kinetic models. Experimental data were used for the pseudo-first order (Eq. 2) [20], the pseudo-second-order (Eq. 3) [21], intraparticular (Eq. 4) [22] and Elovich kinetic models (Eq. 5) construction [23]. Hence, the coefficients of determination (R^2^) of models have indicated the agreement with experimental data.

(2)log(qe-qt)=logqe-k12.303t

(3)tqt=1k2qe2+tqe

(4)qt=kpt0.5+c

(5)qt=1βln(αβ)+1βlnt

### 2.5. Isotherm models

In this study, Langmuir [24] and Freundlich [25] isotherm models were compared to estimate the adsorption manners between adsorbate and adsorbent. Langmuir and Freundlich isotherm models are expressed in Eqs. (6) and (7), respectively.

(6)Ceqe=1qmaxKL+Ceqmax

(7)lnqe=ln(KF)+1nlnCe

where q_e_: adsorbed amount (mg g^–1^), C_e_: adsorbed equilibrium (mg L^–1^), K_L_: Langmuir constant associated with sorption energy (L mg^–1^), q_max_: maximum adsorption capacity (mg g^–1^) relevant to the monolayer coverage, K_F _= Freundlich constant (mg g^–1^), n^–1^: adsorption intensity. 

## 3. Results

### 3.1. Characterization of nanocomposite

**Figure 1:**
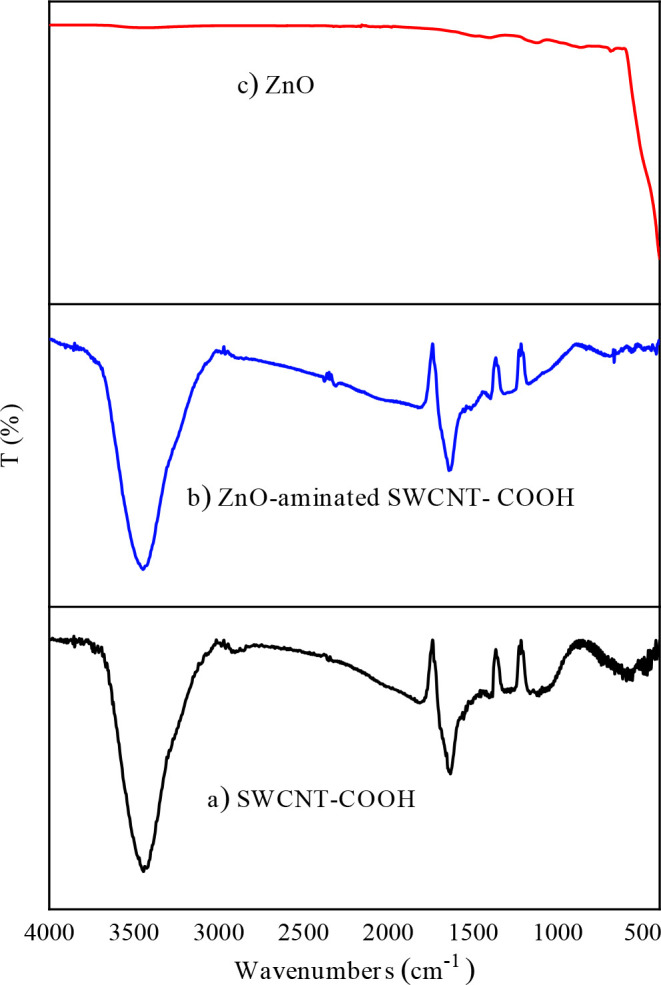
FTIR spectrum of a) SWCNT-COOH b) ZnOaminated SWCNT-COOH c) ZnO.

Three patterns were represented as SWCNT-COOH, ZnO/aminated SWCNT-COOH, and ZnO in Figures 1a-c, respectively. In Figure 1a, a very wide peak appeared in the range 3000–3440 cm^−1^ due to the presence of -OH group [26]. This situation can be explained by dehumidification of SWCNTs-COOH. A small peak appeared at 2950 cm **^–^** ^1^ owing to the C-H stretching [27]. The wavenumber of 1776 cm **^–^** ^1^ ascribed to carbonyl stretching (C=O) vibration [28]. The peak appeared at 1388 cm **^–^** ^1^ owing to the COO- symmetric stretch [29]. In Figure 1 (b), a band with lower frequency emerged at 1562 cm **^–^** ^1^ in the assigned to N-H band [30]. The wavenumbers of 1176 and 1074 cm **^–^** ^1^ are assigned to C–O-C and C-OH bond stretching [31]. ZnO band appeared at the peak of 689 cm **^–^** ^1 ^[32] (Figures 1b-c). The peaks of C-H bond emerged as 871 cm^−1^ and 1123 cm^−1^, respectively [33,34]. Furthermore, at the peak of 1410 cm^−1^, absorbed CO_2_ was examined because of the air atmosphere [35].

**Figure 2:**
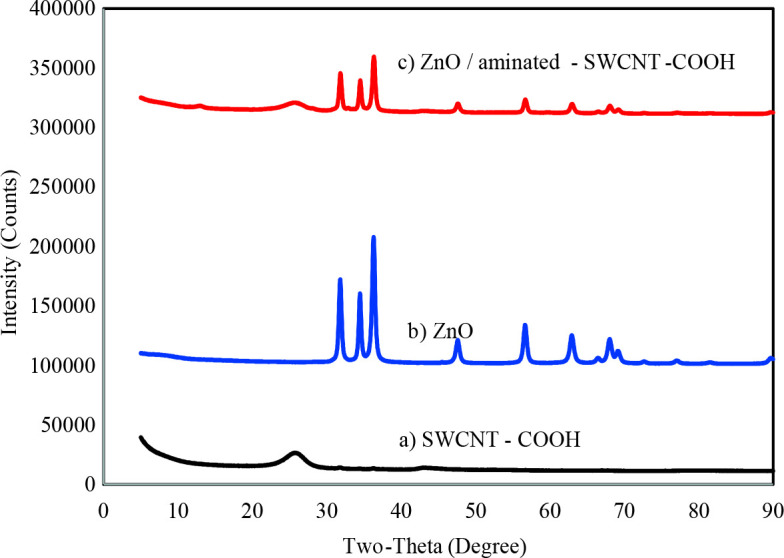
XRD plots a) SWCNT-COOH b) ZnO c) ZnO/aminated SWCNTCOOH.

Three XRD spectrums of SWCNT-COOH, ZnO and ZnO/aminated SWCNT-COOH were represented in Figures 2a-c. A strong peak appeared at 25.65 ^o^ (002) belongs to the graphite sample owing to the hexagonal carbon structure in Figure 2a [36]. Based on Bragg’s law, the interplanar lattice space (d002) was computed as 0.344. Due to the disordered carbon was assigned at the peak of 43.01 ^o^C in Figure 2a [37]. The ZnO nano powder has appeared at 31.82 ˚, 34.47 ˚, 36.31 ˚, 47.60 ˚, 56.66 ˚, 62.94 ˚, 68.03 ˚, and 69.19˚ (Figure 2b). The XRD spectrum of ZnO was well explained according to JCPDS Data Card No: 36-1451. The XRD spectrum of ZnO/aminated SWCNT-COOH was seen in Figure 2c. Low-intensity peak appeared at 25.72 ˚, which refers to the existence of SWCNT-COOH. Using the Debye-Scherrer equation, nanomaterial’s average size has obtained 14 nm.

**Figure 3:**
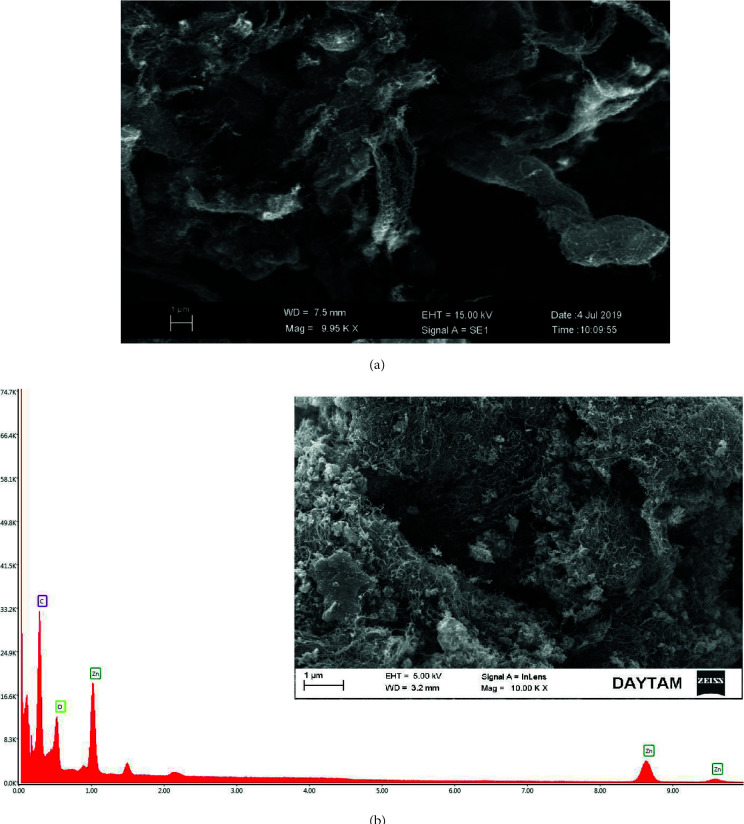
SEM micrograph of a) SWCNT-COOH b) ZnO/aminated SWCNT-COOH with EDX.

Several large pores like ribbons were seen in the SEM graphs belongs to the SWCNT-COOH (Figure 3a) [38]. The surface structure of ZnO/aminated SWCNT-COOH looked like blooming flowers (Figure 3b). ZnO appears to differ from the structure of CNT. Moreover, ZnO disappeared as a set of random directions. It is also in the form of finer quantities of CNT connected by embedded ZnO nanorod. Besides, EDX showed C, O, Zn distributions as 50.90, 30, 19.10, respectively.

### 3.2. Model development

Based on FCCCD and actual data, the predicted values were given in Table 1. Furthermore, statistical experimental error functions were applied to peruse whether the model is compatible or not. The quadratic polynomial equation was acquired in Eq. (8) composed of the single factor, two-factor interactions, and also square interactions of each factor in terms of coded factors. 

**Table 1:** Experimental design constructed by FCCCD with responses.

Run	A:pH	B: Initial concentration	C: adsorbent dose	Adsorption uptake
		(mg L–1)	(mg)	(mg g–1)
1	3	50	15	24.6343
2	5	30	10	36.4667
3	3	10	15	0.66869
4	7	50	15	10.1737
5	5	30	10	36.4818
6	5	10	10	18.2273
7	3	30	10	20.5727
8	7	10	5	16.4606
9	3	10	5	14.4242
10	7	30	10	21.678
11	5	30	5	46.4242
12	5	30	10	36.4758
13	5	30	10	36.4727
14	5	30	15	24.1919
15	7	10	15	5.61616
16	7	50	5	26.6788
17	5	50	10	44.1697
18	5	30	10	36.4818
19	5	30	10	36.4879
20	3	50	5	45.7818

*q* *_MG_* = 35.99 - 2.55 *A* + 9.6 *B* - 8.45 *C* - 5.07 *AB* + 0.9442 *AC* - 1.63 *BC* - 14.12 *A* ^2^ - 4.05 *B* ^2^ + 0.0588 *C* ^2^ (8)

It can be said that the initial concentration of MG and quadratic impact of adsorbent dose has a positive effect on adsorption uptake, at the same time the negative sign parameters also have an antagonistic effect on response.

**Figure 4:**
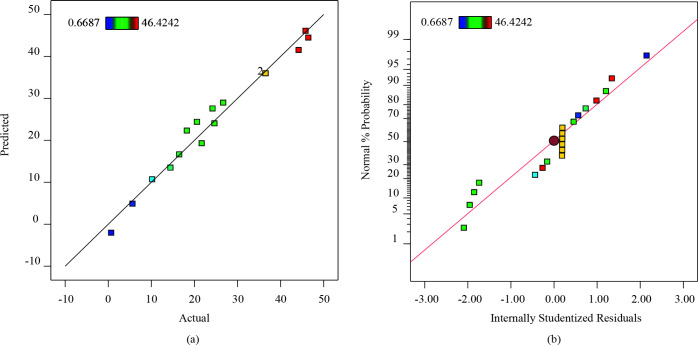
a) predicted versus actual data for MG adsorption onto ZnO/aminated SWCNT-COOH b) the normal % probability versus internally studentized residuals.

Analysis of variance (ANOVA) (Table 2) was done to assess the statistical importance of the model. The determination coefficient of the model (R^2 ^= 0.9780) implied that the model was fitted well for the experimental data. Moreover, the predicted-R^2 ^(0.9581) is very close to adjustment R^2^ (0.8324). This situation represents that a given model is important within the experimental range of factors [39]. The highest influence parameter was seen as the initial concentration of MG due to the higher F value (122.15) and low p-value (< 0.0001). Figure 4a illustrated that predicted versus actual data for MG adsorption onto ZnO-functionalized SWCNTs. Moreover, Figure 4b represented the normal % probability versus internally studentized residuals. This plot demonstrated that error variance was uniform, and the points were not scattered too much. 

**Table 2:** Variance analysis (ANOVA) table for MG adsorption onto ZnO-functionalized SWCNT.

Source	Sum of squares	df	Mean square	F-value	p-value	
Model	3351.34	9	372.37	49.31	< 0.0001	significant
A-pH	64.90	1	64.90	8.59	0.0150	
B-conc	922.39	1	922.39	122.15	< 0.0001	
C-ads	713.77	1	713.77	94.52	< 0.0001	
AB	205.51	1	205.51	27.21	0.0004	
AC	7.13	1	7.13	0.9444	0.3541	
BC	21.30	1	21.30	2.82	0.1240	
A²	548.58	1	548.58	72.64	< 0.0001	
B²	45.12	1	45.12	5.98	0.0346	
C²	0.0095	1	0.0095	0.0013	0.9724	
Residual	75.52	10	7.55			
Pure error	0.0003	5	0.0001			
Corresponding total	3426.86	19				

The Pareto chart was drawn according to Eq. (8) by using the following Eq. (9) [40].

(9)Pi=(bi2∑bi2)x100(i≠ 0)

where b_i _represents the coefficient of factors in Eq (8). the percentage of each factor was identified as P_i_.

**Figure 5:**
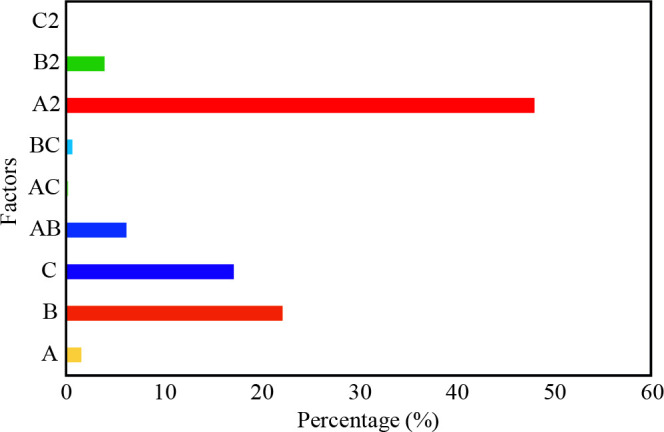
Pareto chart illustrated the influences of factors on response.

Pareto chart illustrated the percentage effects of factors on the adsorption uptake in Figure 5. The square of pH (A^2^, 48.03%) was the most crucial independent, followed by the initial concentration of MG (B, 22.20%). The less importance parameter was found as the square of adsorbent dose (C^2^) under α = 0.05 significant.

### 3.3. Response surface plots with contour graphs

**Figure 6:**
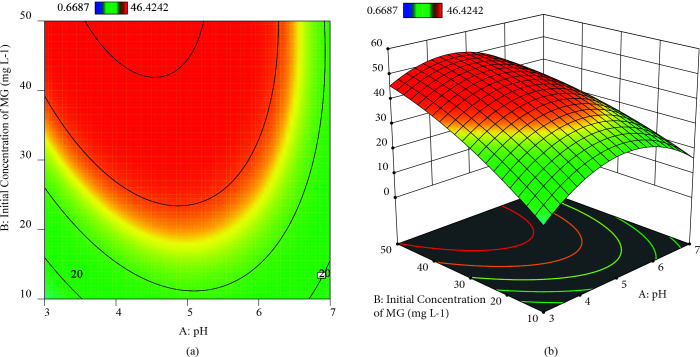
a) contour plots of dual interaction of initial concentration of MG and pH on adsorption uptake b) 3-D plots of dual interaction of initial concentration of MG and pH on adsorption uptake.

Generating 3D graphs with contour graph (2D) by using Design Expert package program (Trial Version 12) were represented in Figures 6 **–** 8 for the investigation of the predicted model. The optimal factors with the maximal response were seen in these graphs. Besides, three-dimensional graphics help us understand the relationship between response and factors. 

**Figure 7:**
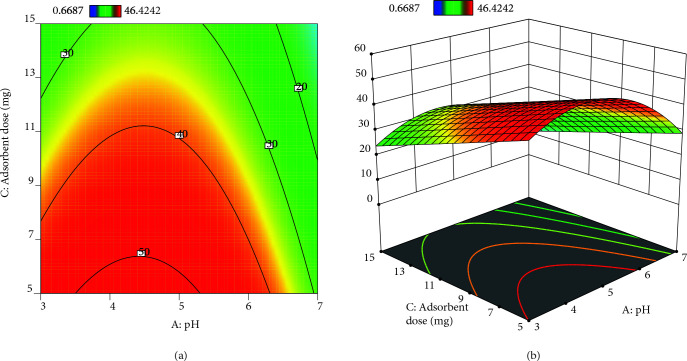
a) contour plots of dual interaction of adsorbent dose and pH on adsorption uptake b) 3-D plots of dual interaction of adsorption dose and pH on adsorption uptake.

If the synergistic relationship between two variables is important, the shape of the contour graphic is a saddle, otherwise, its shape is circular [41]. For MG adsorption onto ZnO-functionalized SWCNTs, the optimal response was written as a value in the flag on the contour zone in Figures 6a and 6b. When pH value was 4.63 and initial concentration of MG was 49.94 mg L **^–^** ^1^, adsorption uptake of adsorbent was increased up to the 52.26 mg g **^–^** ^1^. The adsorption uptake was enhanced from 18.54 to 44.17 mg g **^–^** ^1^ when the initial concentration was raised from 10 to 50 mg L **^–^** ^1^. The increase in concentration increases the interaction between the dye and the adsorbent, except to provide the driving force required to control resistance to dye and mass transfer. Therefore, with an increment in dye concentration, the adsorption rate and hence dye uptake increased [42]. The maximum adsorption uptake was observed at pH 4.63 in Figures 6a and 6b. As known, MG is a cationic dye, so it gives a positive charge in distilled water. At pH 3, there are a large number of protonated ions on the adsorbent surface may repel the cationic MG, which leads to a lower adsorption uptake. With increasing pH up to 4.63, the surface charge of the adsorbent comes to more negative. As a result, the stronger electrostatic interaction happens between adsorbate and adsorbent [43]. Besides, complete ionization occurred at pH < 4 or pH 4 [42,44]. In Figures 7a and 7b, 5.25 mg of adsorbent dose and pH 4.63 increased the adsorption uptake. But, 3D graph indicated that the adsorbent dose was uncritically influenced on the dye adsorption. In order to get higher adsorption uptake, it was unessential to study at a higher dosage. Adsorption uptake was decreased from 52.26 to 24.63 mg **^–^** ^1^ to as the amount of adsorbent rose from 5 to 15 mg in the MG solution. This situation is clearly explained since the adsorption zone overlaps because of excessive crowding clustering of the amount of adsorbent [45].

**Figure 8:**
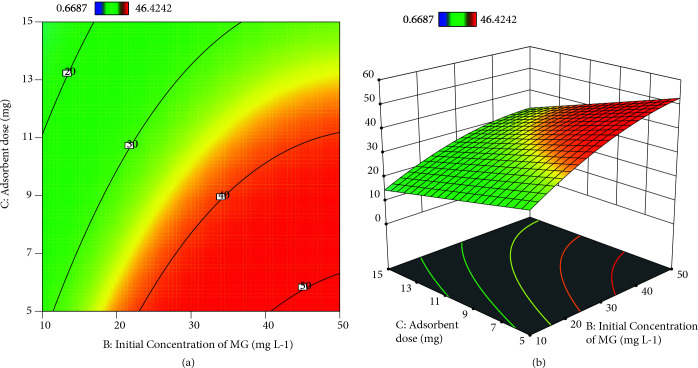
a) contour plots of dual interaction of adsorbent dose and initial concentration of MG on adsorption uptake b) 3-D plots of dual interaction of adsorbent dose and initial concentration of MG on adsorption uptake.

Under the 5.25 mg of adsorbent dose and 49.94 mgL **^–^** ^1^ initial concentration of MG, the highest adsorption uptake was obtained in Figures 8a and 8b. 

Moreover, the effect of each material on adsorption capacity of MG was observed at optimal conditions. Table 3 showed their adsorption capacity. The SWCNT-COOH exhibited better adsorption capacity on MG. The synthesized nanomaterial had a 15% higher adsorption capacity than pure ZnO. 

**Table 3:** The comparison of MG adsorption capacity of ZnO, SWCNT-COOH, and ZnO/aminated SWCNT-COOH.

Material	Adsorption capacity (mg g–1)
ZnO	42.26
SWCNT-COOH	50.92
ZnO/aminated SWCNT-COOH.	49.21

### 3.4. Adsorption kinetics

**Figure 9:**
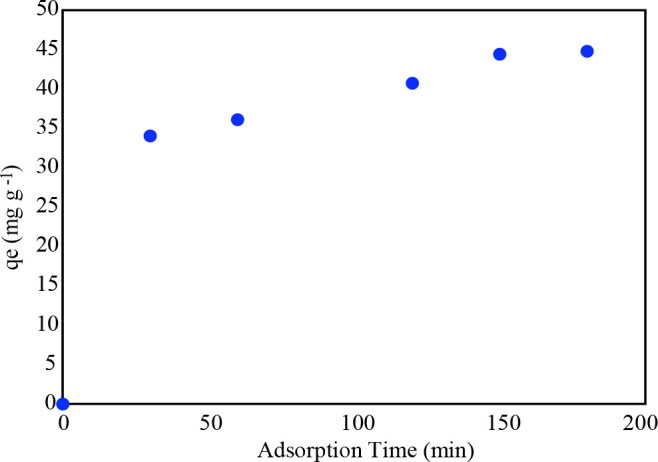
The influence of contact time on the adsorption capacity of MG onto the ZnO/aminated SWCNT-COOH.

In order to the wastewater treatment for industrial application, it is important to determine the equilibrium time. The influence of contact time was seen in Figure 9. The sharp increment was indicated up to 30 min, after the 180 min later, the adsorption capacity was reached in equilibrium. Seventy-six % of the adsorption capacity obtained at the equilibrium time was reached within 30 min. At first stage, a very sudden increase of adsorption capacity was observed due to the vacant sites of adsorbent. At second stage, the adsorbed active groups with MG molecule, after a while, they reached a saturation. Thus, diffusion was no longer formed from the bulk phase through the adsorbent interface.

Table 4 represented the coefficient of kinetic models. It can be said that the excellent fitting model with the highest determination coefficient was obtained (R^2 ^= 0.9912) in pseudo-second-order kinetic. Table 4 indicated the MG adsorption onto the ZnO/aminated SWCNT-COOH nanocomposites satisfied with the pseudo-second-order model. When the intra-particle diffusion model was examined, it appears that the plot’s straight is linear and goes through the origin. So, the sole rate-limiting procedure has occurred.

**Table 4:** Applied kinetic models with the coefficients of parameters.

Models	Parameters	Coefficients
Pseudo-first-order	qe (exp)qe (model) k1R2	44.8030.100.01870.9420
Pseudo-second-order	qe (model)k2R2	45.250.00270.9912
Elovich	αβR2	9.79350.11650.9858
Intraparticular	KpCR2	3.19447.12380.8545

### 3.5. Adsorption isotherms

The results, according to the four isotherm models, were indicated in Table 5. The highest R^2^ value belongs to the Freundlich isotherm model, which suggested the Freundlich isotherm model was well-fitted to the adsorption process. Thus, the adsorption of MG adsorption onto the ZnO/aminated SWCNT-COOH was inclined to multiple heterogeneous layers.

**Table 5:** Applied isotherm models with the coefficients of parameters.

Models	Parameters	Coefficients
Langmuir	qe (model)kLR2	60.610.01880.9372
Freundlich	nkFR2	1.4041.87700.9791

### 3.6. Thermodynamic parameters

The following Eqs. (10–12) represent the thermodynamics parameters such as Gibbs free energy change (ΔG°), enthalpy (ΔH°), and entropy (ΔS°).

∆G = - RT lnKads (10) 

Kads = qe/C_e_ (11)

∆G = ∆H - T∆S (12) 

where T is the absolute temperature (K) of solution, R is the universal gas constant (8.314 J/mol. K). Kad represents the single point and is calculated as qe/Ce. The tangent of the plot of ln (qe/Ce) vs. 1/T represents the –ΔH°/R. Furthermore, the intercept of the graph indicates ΔS° /R. 

**Figure 10:**
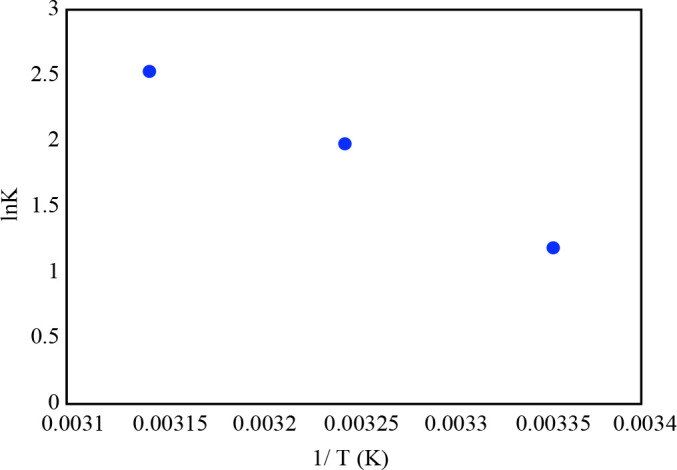
The graph of lnK vs. 1/T.

Figure 10 represented the lnK vs. 1/T. Table 6 indicated the thermodynamic parameters for MG adsorption onto ZnO/aminated SWCNT-COOH. The positive value of ΔH° expresses the endothermic process. The negative sign of ΔG° represents spontaneous process. The ΔS^o^ of ZnO/aminated SWCNT-COOH adsorbing MG dye was higher than 0, which means that the disorder of adsorption process. The calculated ΔH° as an endothermic process that adsorbed energy as heat in the system is assignable chemisorption [46].

**Table 6:** The calculated the thermodynamic parameters for MG adsorption onto ZnO/aminated SWCNT-COOH.

T (K)	ΔG° (KJ/mol)	ΔH°(KJ/mol)	ΔS°(J/mol)
298.15	–2.95	53.02	187.99
313.15	–4.91		
323.15	–6.27		

## 4. Discussion

Briefly, the ZnO-functionalized SWCNTs were successfully synthesized. The prepared material was used for the evacuation of MG as a model dye. Furthermore, the structure elucidation of the adsorbent was determined using various characterization techniques such as FTIR, XRD, and SEM-EDX. The surface shape of the nanocomposite was nanoflower. FCCCD optimization was done by selecting three variables such as MG concentration, pH and adsorbent dose. The determination of the coefficient (R^2 ^= 0.9780) implied that the model is significantly fit the experimental data. Besides, the Pareto chart indicated that the square of pH was the most important variable among the other variables. Optimization of the adsorption uptake by RSM based on FCCCD revealed the maximal adsorption uptake was determined as 52.26 mg g^–1^ under the optimal conditions (4.63 for pH, 49.94 mg L^–1^ for initial concentration, 5.25 mg for adsorbent dose). Moreover, kinetic data were in accordance with the pseudo second order (R^2 ^= 0.9912). Besides, the Freundlich isotherm model (R^2 ^= 0.9791) is well-explained the adsorbent-adsorbate behaviour.
